# Radiomic Evaluations of the Diagnostic Performance of DM, DBT, DCE MRI, DWI, and Their Combination for the Diagnosisof Breast Cancer

**DOI:** 10.3389/fonc.2021.725922

**Published:** 2021-09-10

**Authors:** Shuxian Niu, Xiaoyu Wang, Nannan Zhao, Guanyu Liu, Yangyang Kan, Yue Dong, E-Nuo Cui, Yahong Luo, Tao Yu, Xiran Jiang

**Affiliations:** ^1^Department of Biomedical Engineering, School of Fundamental Sciences, China Medical University, Shenyang, China; ^2^Liaoning Cancer Hospital and Institute, Cancer Hospital of China Medical University, Shenyang, China; ^3^School of Computer Science and Engineering, Shenyang University, Shenyang, China

**Keywords:** breast, mammography, MRI, radiomics, nomogram

## Abstract

**Objectives:**

This study aims to evaluate digital mammography (DM), digital breast tomosynthesis (DBT), dynamic contrast-enhanced (DCE), and diffusion-weighted (DW) MRI, individually and combined, for the values in the diagnosis of breast cancer, and propose a visualized clinical-radiomics nomogram for potential clinical uses.

**Methods:**

A total of 120 patients were enrolled between September 2017 and July 2018, all underwent preoperative DM, DBT, DCE, and DWI scans. Radiomics features were extracted and selected using the least absolute shrinkage and selection operator (LASSO) regression. A radiomics nomogram was constructed integrating the radiomics signature and important clinical predictors, and assessed with the receiver operating characteristic (ROC) curve, calibration curve, and decision curve analysis (DCA).

**Results:**

The radiomics signature derived from DBT plus DM generated a lower area under the ROC curve (AUC) and sensitivity, but a higher specificity compared with that from DCE plus DWI. The nomogram integrating the combined radiomics signature, age, and menstruation status achieved the best diagnostic performance in the training (AUCs, nomogram *vs.* combined radiomics signature *vs.* clinical model, 0.975 *vs.* 0.964 *vs.* 0.782) and validation (AUCs, nomogram *vs.* combined radiomics signature *vs.* clinical model, 0.983 *vs.* 0.978 *vs.* 0.680) cohorts. DCA confirmed the potential clinical usefulness of the nomogram.

**Conclusions:**

The DBT plus DM provided a lower AUC and sensitivity, but a higher specificity than DCE plus DWI for detecting breast cancer. The proposed clinical-radiomics nomogram has diagnostic advantages over each modality, and can be considered as an efficient tool for breast cancer screening.

## Introduction

Breast cancer has been a major concern and the second leading cause of cancer death among women (1). The prevalence of breast cancer has increased in the recent years, mainly due to the implementation of an early screening mammography (2). Although there is still no effective way to prevent breast cancer, studies have shown that early detection and treatment can increase the chance of full recovery for the patients (3).

Digital mammography (DM) using 2D technique, as a widely used tool for detecting breast cancer, has a serious limitation that the visibility of lesions may be decreased since they are frequently obscured by dense fibroglandular and other normal tissues within the breast (4), which often leads to a missed diagnosis or misdiagnosis (5). To address this issue, digital breast tomosynthesis (DBT) rotates the X-ray tubes in a limited angle, thus allowing an improved identification of anomalies obscured by normal tissues (6, 7). Therefore, the DBT is commonly considered to be capable of decreasing the recall rates and increasing the detection rates for breast cancer compared with DM (8). Magnetic resonance imaging (MRI), as another popular tool for breast screening, has been demonstrated to be very sensitive in detecting breast cancer (9). While, the relative low specificity of MRI screening may lead to a high rate of overtreatment (10). Besides, the high examination fees of MRI also hinder the clinical application in early breast screening.

In the clinical practice, the diagnosis of breast cancer based on DM, DBT, or MRI mainly relies on visual inspections of the morphological changes of breast lesions, including size, shape, and gray level changes, and, thus, require experienced clinicians to make decisions. Previous reports have compared the diagnostic capabilities of DM with DBT (11, 12) and mammography with MRI (13, 14), all based on subjective visual examinations and the lack of quantified assessments. Recently, the radiomics-based computer aided diagnosis (CAD) has received increasing attention due to its quantitative advantages (15, 16). By using automated data characterization algorithms, the radiomics can extract and select discriminative and quantified features from a region of interest, which were shown to reflect biological information regarding the tumor and were highly correlated with disease status (17). Subsequent analysis, including statistics, machine learning classifiers, and nomogram can give associations between imaging features and the underlying pathophysiology (18). Radiomics-based studies on breast cancer have been proposed for predicting the axillary lymph node metastasis (19–23), molecular subtypes (24–28), tumor grades (29–31), and treatment responses (32–37). Some recent studies also conducted a radiomics-based quantified analysis for the diagnosis of breast cancer based on DM (38, 39), DBT (40, 41), and MRI (42, 43) separately, and demonstrated improvements of the diagnostic performance using radiomics compared with visual examinations by radiologists. A recent effort evaluated T2W, DCE, and DWI separately and in combination, but ignored the clinical values of mammography screening, and lack of correlating their findings with clinical evaluation, which may limit the clinical applicability (44).

To our knowledge, direct and quantified comparisons among MD, DBT, and MRI have not been reported. Therefore, the present study aims to widen the understanding of mammography and MRI in breast cancer screening by directly and quantitatively comparing the diagnostic efficiency of each modality individually and in combination. Besides, this study aims to propose a visualized clinical-radiomics nomogram based on the optimal imaging combination and important clinical factors for early assessment of suspected breast lesions.

## Material and Methods

### Patients

This retrospective analysis of breast DM, DBT, and MRI data was approved by the Institutional Research Ethics Board of our institute (Approval No. 2013010). The informed consent requirement was waived. A total of 120 patients [mean age ± standard deviation (SD), 48.81 ± 10.83] were enrolled between September 2017 and July 2018 in our hospital. The number of the patients harboring pathologically confirmed benign or malignant lesions were 50 and 70, respectively. Inclusion criteria were as follows: (i) older than 18 years; (ii) underwent DM, DBT, and MRI screening before surgery; and (iii) underwent surgical resection with pathological confirmation. Exclusion criteria were: (i) combined with other tumor diseases; (ii) during menstruation, pregnancy, or lactation periods; (iii) history of breast surgery, radiotherapy, or chemotherapy, as well as breast implants; and (iv) having artifacts in the images. All patients were randomly divided into training and validation cohorts at a 2:1 ratio using stratified sampling. Clinical factors including age, family history of breast cancer, history of biopsy, and menstruation status were obtained from the electronic medical record system of our hospital.

### Digital Mammography, Digital Breast Tomosynthesis, and Magnetic Resonance Imaging Acquisitions 

Preoperative DM and DBT examinations were performed by a radiographer with 10 years of work experience using a DBT scanner (Hologic Selenia Dimensions, Hologic, USA). The obtained images of the compressed breast were reconstructed with a 1-mm intersection spacing to give a three-dimensional view of the tissue, slice by slice, and suitably spaced. The number of the slices depends on the compressed breast thickness. The following parameters were used to perform the DBT scanning: The voltage range of the X-ray tubes: 20.0–49.0 kV (step: 1.0 kV), nominal power: 3.0 kW, current time range: 300–400 mAs, scanning time < 4.0 s, reconstruction time: 2.0–5.0 s, and pixel size: 70 μm. The obtained DBT images were interpreted on a Hologic breast computer-aided diagnosis (CAD) workstation (SecureViewDx; Hologic) equipped with two 5-megapixel monitors.

Preoperative MRI scans were performed using a 1.5-T MRI scanner (HDx, GE Healthcare). The axial diffusion-weighted imaging was used with the following parameters: the b-value: 800 s/mm^2^, repetition time (TR)/echo time (TE)/inversion time (TI): 5,000 ms/64 ms/0 ms, flip angle: 90°, slice thickness: 6 mm, slice gap: 7.5 mm, field of view: 240 mm, matrix size: 128 × 128. The axially vibrant sequence (a 3D T1-weighted imaging technique covering bilateral breasts conventional scans or dynamic enhanced scans to obtain axial or sagittal images with high signal-to-noise ratio and high resolution) with the following parameters: TR/TE/TI: 6.2 ms/3.0 ms/13 ms; flip angle: 10°; slice thickness: 3.2 mm; slice gap: 3.2 mm, 48 slices per volume; field of view: 360 mm; matrix size: 350 × 350. The contrast agent was injected intravenously (0.1 mmol/kg of Gd-DTPA-MBA, Omniscan, GE Healthcare), followed by a 20-mL saline flush, both at the rate of 3 ml/s. After the intravenous injection, continuous non-interval scans were performed in eight phases, with a scan time for each phase of 43 seconds. All scanned images were stored in the Picture Archiving and Communication System (PACS) in our hospital in a Digital Imaging and Communications in Medicine (DICOM) format. The details about their scan parameters are shown in **Supplementary Tables S1, S2**.

### Breast Lesion Segmentation

Regions of interest (ROIs) were manually segmented slice by slice for each patient using the ITK-SNAP software (version 3.6.0) by a radiologist with 12 years of working experience according to the breast imaging reporting and data system (BI-RADS). The radiologist was blinded to the pathological results for the patients. The ROIs included the breast lesions and edges, exporting as a compressed package in an NII format for further analysis.

### Radiomics Feature Extraction

Radiomics features including 18 first-order statistical, 13 shape-based, and 74 textual features were extracted based on the segmented ROIs using the Pyradiomics package in Python 3.6 (https://pyradiomics.readthedocs.io/en/). The texture feature category consists of the gray level cooccurence matrix (GLCM), gray level run length matrix (GLRLM), gray level size zone matrix (GLSZM), neighboring gray tone difference matrix (NGTDM), and gray level dependence matrix (GLDM) features. The first-order and texture features were also calculated from the original images that were filtered with eight types of filters: logarithm, square, gradient, exponential, laplacian of Gaussian, wavelet, and localbinarypattern2D (45). Detailed descriptions of the features and calculation protocols can be found in a previous report (46).

### Feature Selection

To obtain reliable and discriminative features, 30 patients were randomly selected to perform the intraclass correlation coefficient (ICC) analysis (47), 15 from the training group and 15 from the validation group. The ROIs were double-blind segmented by another radiologist with 8 years of working experience. Features with ICC > 0.75 were retained, then further selected by the Mann-Whitney *U* test. Features with *P *< 0.05 were considered significant variables between the benign and malignant groups. Finally, the least absolute shrinkage and selection operator (LASSO) logistic regression was used to identify the most discriminative features with a 10-fold cross-validation for selecting the parameter lambda using the “glmnet” package in R language v3.6 (available from URL: https://www.r-project.org) (48).

### Development of the Radiomics Signature, Clinical Model, and Nomogram 

The radiomics signature formula was calculated for each patient by a linear combination of the selected features weighted by the respective LASSO coefficients. The logistic regression was used to identify the discriminative clinical predictors. A clinical model was established using the multivariate logistic regression with the Akaike’s Information Criterion (AIC) as the stopping rule (49). A radiomics nomogram for differentiating benign and malignant lesions was constructed incorporating the radiomics signature and the most important clinical factors using the “rms” package in R v.3.6.

### Statistical Analysis

The Mann-Whitney *U*-test, t-test, Chi-Square test, and Shapiro-Wilk test were performed on continuous and discrete variables, respectively. All hypothesis tests were two-sided. The ROC curve analysis was performed to evaluate the diagnostic performance of each model, with the area under the ROC curve (AUC), accuracy, sensitivity, and specificity calculated as comparison metrics. The optimal cutoff value was obtained on the ROC curve with the maximum Youden index (50). ROC curves were evaluated with the DeLong test using the “pROC” package in R. Calibration curves were plotted to assess the calibration of the model-predicted results with truth values. The decision curve analysis (DCA) (51) was performed using the “rmda” package to assess the potential clinical usefulness of the models.

## Results

### Patient Characteristics

The clinical characteristics of the patients were statistically analyzed and shown in **Table 1**. The age and menstruation status were significantly different between the benign and malignant groups (*P *< 0.05). No statistical difference was observed in the types of family history and history of biopsy. A clinical model was built integrating the age and menstruation status for detecting malignant lesions.

**Table 1:** >Statistical analysis results of clinical characteristics.

Characteristic	Training cohort		*P*	Validation cohort		*P*
	Benign (n = 33)	Malignant (n = 46)		Benign (n = 17)	Malignant (n = 24)	
Age (years)			0.008			0.009
<40	10 (30.3)	4 (8.7)		6 (35.2)	2 (8.3)	
40–49	14 (42.4)	19 (41.3)		8 (47.1)	9 (37.5)	
50–59	8 (24.2)	11 (23.9)		3 (17.6)	4 (16.7)	
>=60	1 (3.0)	12 (26.1)		0 (0.0)	9 (37.5)	
Family history of breast cancer, n (%)			0.693			1.000
+	2 (6.1)	5 (10.9)		2 (11.8)	2 (8.3)	
–	31 (93.9)	41 (89.1)		15 (88.2)	22 (91.7)	
History of biopsy, n (%)			0.171			1.000
+	2 (6.1)	0 (0.0)		1 (89.7)	1 (4.2)	
–	31 (93.9)	46 (1.0)		16 (10.3)	23 (95.8)	
Menstruation status, n (%)			0.001			0.002
+	6 (76.8)	20 (49.3)		1 (89.7)	13 (30.3)	
–	27 (23.2)	26 (50.7)		16 (10.3)	11 (69.7)	
BI-RADS (DM plus DBT), n (%)			<0.001			<0.001
0, 1, 2, 3	8 (24.2)	0 (0.0)		6 (35.3)	1 (4.2)	
4A, 4B, 4C	24 (72.7)	32 (69.6)		11 (64.7)	15 (62.5)	
5, 6	1 (3.0)	14 (30.4)		0 (0.0)	8 (33.3)	
BI-RADS (MRI), n (%)						<0.001
1, 2, 3	18 (54.5)	0 (0.0)		8 (47.1)	0 (0.0)	
4, 5	15 (45.5)	46 (100.0)		9 (52.9)	24 (100.0)	

BI-RADS, breast imaging reporting and data system; DM, digital mammography; DBT, digital breast tomosynthesis; MRI, magnetic resonance imaging.

### Evaluation of Diagnostic Performance of Digital Mammography, Digital Breast Tomosynthesis, and Magnetic Resonance Imaging

**Figure 1:**
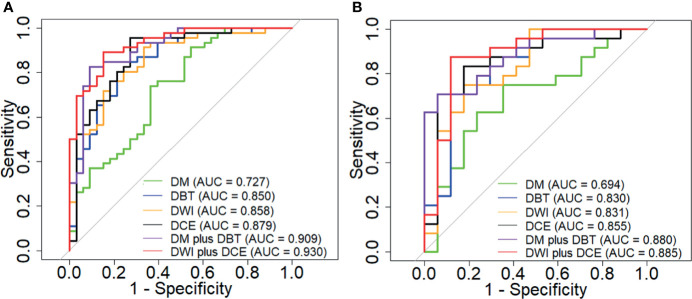
ROC curves of the DM, DBT, DCE MRI and DWI MRI used individually and in comibination in the training **(A)** and validation **(B)** cohort.

Diagnostic performance of the radiomics signature derived from the DM, DBT, DCE, and DWI individually and in combination were assessed (**Table 2**). **Figure 1** shows the ROC curves of each radiomics signature. The results indicated that the DCE generated the highest AUCs and sensitivities among the four modalities, but had relatively low specificities. The diagnostic performance of DWI plus DCE was significantly higher than DM plus DBT in terms of sensitivity. Besides, the DWI plus DCE yielded the highest positive predictive values (PPV) and the lowest misdiagnosis rates.

**Table 2:** Diagnostic performance of each modality used alone and in combination.

	Cohort	AUC(95%CI)	ACC (95%CI)	SEN	SPE	PPV	NPV
DM alone	Training Cohort	0.727 (0.612–0.842)	0.696 (0.583–0.795)	0.739	0.636	0.739	0.636
	Validation Cohort	0.694 (0.524–0.863)	0.707 (0.545–0.839)	0.750	0.647	0.750	0.647
DBT alone	Training Cohort	0.850 (0.766–0.940)	0.798 (0.692–0.880)	0.804	0.788	0.841	0.743
	Validation Cohort	0.830 (0.698–0.968)	0.781 (0.624–0.894)	0.708	0.882	0.895	0.682
DWI MRI	Training Cohort	0.858 (0.775–0.942)	0.810 (0.706–0.890)	0.913	0.667	0.793	0.846
	Validation Cohort	0.831 (0.696–0.966)	0.781 (0.624–0.894)	0.750	0.824	0.857	0.700
DCE MRI	Training Cohort	0.879 (0.978–0.960)	0.861 (0.765–0.928)	0.957	0.727	0.830	0.923
	Validation Cohort	0.855 (0.727–0.984)	0.829 (0.674–0.929)	0.833	0.824	0.870	0.778
DM plus DBT	Training Cohort	0.909 (0.842–0.976)	0.861 (0765–0.928)	0.826	0.909	0.927	0.790
	Validation Cohort	0.880 (0.779–0.981)	0.805 (0.651–0.912)	0.708	0.941	0.944	0.700
DWI plus DCE	Training Cohort	0.930 (0.877–0.982)	0.873 (0.780–0.938)	0.891	0.849	0.891	0.849
	Validation Cohort	0.885 (0.768–1.000)	0.878 (0.738–0.959)	0.875	0.882	0.913	0.833

DM, digital mammography; DBT, digital breast tomosynthesis; DWI, diffusion-weighted imaging; DCE, dynamic contrast enhanced; AUC, area under the ROC curve; CI, confidence interval; Acc, accuracy; Sen, sensitivity; Spe, specificity; PPV, positive predictive value; NPV, negative predictive value.

### Development of the Combined Radiomics Signature and Nomogram

Radiomics features selected from the four modalities were combined and further selected to generate a combined feature set consisting of seven features, three from DBT, two from DCE, and two from DWI. Diagnostic performance of each feature was evaluated and is listed in **Table 3**. The combined radiomics signature (combined Rad score, **Supplementary S3**.) integrating the seven features and their corresponding LASSO coefficients was built and shown as follows: 


Combined Rad score=0.5665−Wavelet_HHL_glszm_ZonePercentage×2.7374+ Wavelet_LHL_firstorder_Skewness×1.4977+ Log_sigma_3_0_mm_3D_glrlm_ShortRunLowGrayLevelEmphasis×2.1381+ Wavelet_HHLglcm_Imcl×1.8133+Original_glcm_ClusterShade×1.4596+ Logarithm_glcm_InverseVariance×1.6268−Exponential_glcm_MCC×0.7365.

**Table 3:** Diagnostic performance of the selected features for the diagnosis of breast lesions.

Feature	Dataset	Mean ± SD		*P*-value	AUC
		Benign	Malignant		
Wavelet_HHL_glszm_ZonePercentage	Training Cohort	0.006 ± 0.006	0.002 ± 0.002	<0.001	0.772
	Validation Cohort	0.006 ± 0.006	0.002 ± 0.002	0.021	0.716
Wavelet_LHL_firstorder_Skewness	Training Cohort	0.076 ± 0.297	-0.147 ± 0.215	<0.001	0.737
	Validation Cohort	0.040 ± 0.377	-0.176 ± 0.168	0.010	0.740
Log_sigma_3_0_mm_3D_glrlm_ShortRunLowGrayLevelEmphasis	Training Cohort	0.057 ± 0.028	0.037 ± 0.020	<0.001	0.736
	Validation Cohort	0.062 ± 0.062	0.035 ± 0.015	0.181	0.625
Wavelet_HHLglcm_Imcl	Training Cohort	-0.099 ± 0.049	-0.072 ± 0.033	<0.001	0.738
	Validation Cohort	-0.085 ± 0.042	-0.067 ± 0.015	0.181	0.625
Original_glcm_Clus-terShade	Training Cohort	-2,413.833 ± 11,596.710	3,361.392 ± 14,159.810	0.026	0.648
	Validation Cohort	-2,950.967 ± 10,227.370	5,047.669 ± 1,264.26	0.013	0.730
Logarithm_glcm_InverseVariance	Training Cohort	0.161 ± 0.026	0.146 ± 0.022	<0.001	0.667
	Validation Cohort	0.152 ± 0.022	0.151 ± 0.020	<0.001	0.507
Exponential_glcm_MCC	Training Cohort	0.583 ± 0.305	0.776 ± 0.158	0.002	0.710
	Validation Cohort	0.539 ± 0.269	0.761 ± 0.155	0.003	0.772

Glszm, gray level size zone matrix; glrlm, gray level run length matrix; glcm, gray level co-occurrence matrix; SD, standard deviation; AUC, area under the ROC curve.

**Figure 2:**
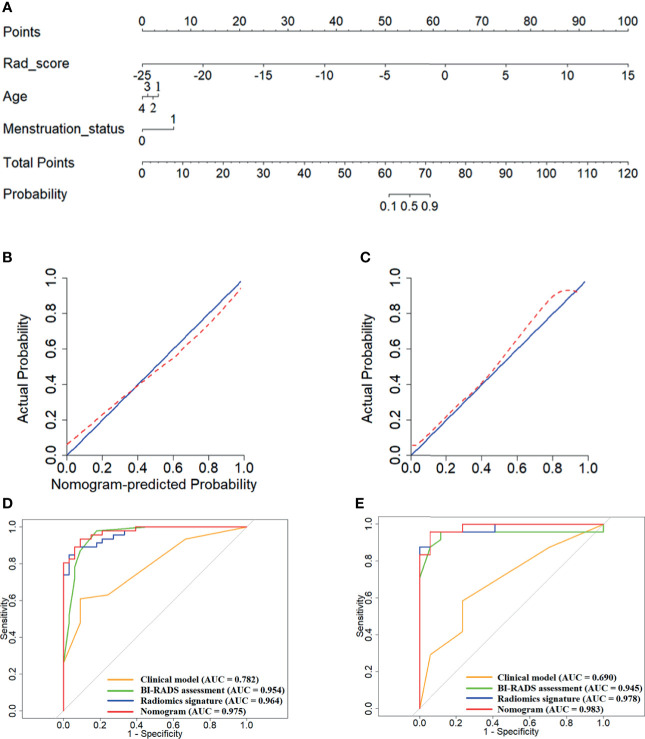
Development and validation of the nomogram model integrating the combined Rad score, age and menstruation status. **(A)** Construction of the nomogram; **(B, C)**, Calibration curves of the nomogram in the training **(B)** and validation **(C)** cohort; **(D, E)**, ROC curves of the nomogram, combined Rad score and clinical model in the training **(D)** and validation **(E)** cohort.

A radiomics nomogram was constructed integrating the combined Rad score with the age and menstruation status (**Figure 2A**). The risk of being a malignant lesion can be read off the scale in the last row by vertically drawing a line from the total points. Calibration curves are shown in **Figures 2B, C**, indicating acceptable agreements between the nomogram-estimated probabilities and actual outcomes of the lesions. The 45-degree blue line and the red dotted line represent an ideal diagnosis and the performance of our nomogram, respectively. As the red dotted line is closer to the blue line represents a better diagnostic performance. **Figures 2D, E** show that the nomogram exhibited better diagnostic capabilities compared with the combined Rad score or the clinical model alone (AUCs in the training cohort, nomogram *vs.* combined Rad score *vs.* clinical model, 0.975 *vs.* 0.964 *vs.* 0.782; AUCs in the validation cohort, nomogram *vs.* combined Rad score *vs.* clinical model, 0.978 *vs.* 0.983 *vs.* 0.690). The diagnostic performance of the combined Rad score, clinical model and nomogram are shown in **Table 4**.

**Table 4:** Comparison of the clinical model, BI-RADS assessment, combined Rad score, and nomogram.

	Training cohort							Validation cohort						
	AUC (95% CI)	Acc (95% CI)	Sen	Spe	PPV	NPV	*P*	AUC (95% CI)	Acc (95% CI)	Sen	Spe	PPV	NPV	*P*
M1	0.782 (0.687–0.877)	0.734 (0.623–0.827)	0.609	0.909	0.903	0.625		0.690 (0.531–0.849)	0.659 (0.494–0.799)	0.583	0.765	0.778	0.565	
M2	0.954 (0.908–1.000)	0.912 (0.826–0.964)	0.978	0.818	0.883	0.964		0.945 (0.861–1.000)	0.927 (0.801–0.985)	0.958	0.882	0.920	0.938	
M3	0.964 (0.931–0.997)	0.911 (0.826–0.964)	0.935	0.909	0.935	0.909		0.978 (0.941–1.000)	0.951 (0.835–0.994)	0.958	0.941	0.958	0.941	
M4	0.975 (0.948–1.000)	0.924 (0.842–0.972)	0.913	0.849	0.894	0.875		0.983 (0.955–1.000)	0.951 (0.835–0.994)	0.958	0.941	0.958	0.941	
M1 *vs.* M2							<0.001							<0.001
M2 *vs.* M3							0.741							0.404
M3 *vs.* M4							0.178							0.596
M1 *vs.* M4							0.001							<0.001

M1, Clinical model; M2, BI-RADS assessment; M3, Combined Rad score; M4, Nomogram model; AUC, area under the ROC curve; CI, confidence interval; Acc, accuracy; Sen, sensitivity; Spe, specificity; PPV, positive predictive value; NPV, negative predictive value.

**Figure 3:**
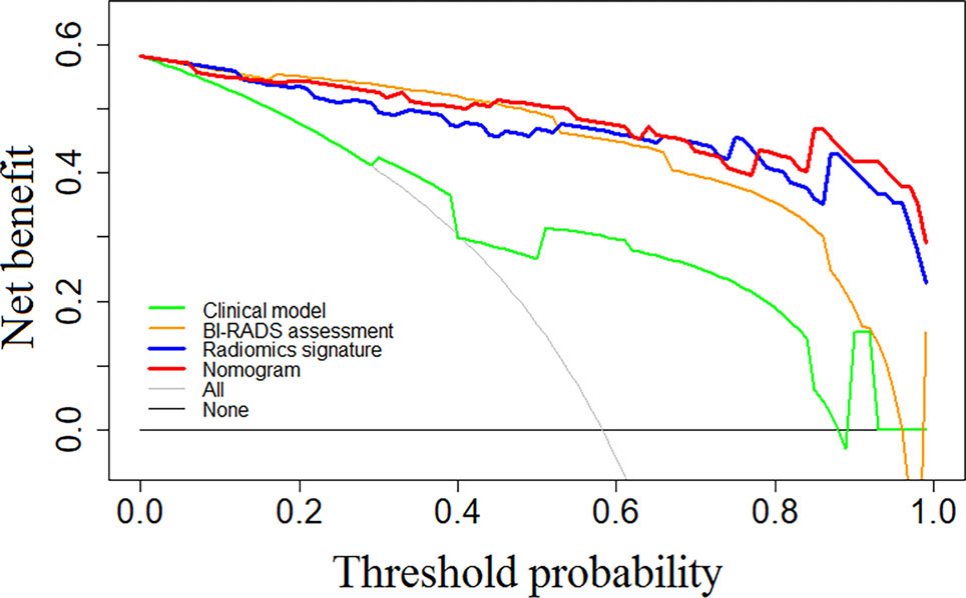
Showed results of the decision curve analysis for each model. The nomogram exhibited greater net benefit compared with the combined Rad score or the clinical model. When the threshold probability of the patient was between 0.44 and 0.68, or over 0.78, greater benefit can be obtained by using the nomogram, indicating good potential in clinical applications.

**Figure 3** shows the results of the decision curve analysis for each model. The nomogram exhibited a greater net benefit compared with the combined Rad score or the clinical model. When the threshold probability of the patient was between 0.44 and 0.68, or over 0.78, a greater benefit can be obtained by using the nomogram, indicating a good potential in clinical applications.

## Discussion

Prior to this study, there have been researches evaluating the diagnostic capabilities of DM (32, 38, 39), DBT (40, 41), MRI (42–44) separately for detecting breast cancer, all based on subjective visual examinations, and lack of direct and quantitative comparisons of different modalities. On the contrary, this study performed comprehensive radiomics analyses to quantitatively assess the diagnostic performance of different modalities separately and in combination. We found that the radiomics signature derived from DM always showed the worst diagnostic performance in terms of AUC, sensitivity, and specificity compared with the other individual modalities. This may be explainable since the DM only obtains one image, which may lead to overlapping glands, and, hence, is not sufficient to analyze the distribution of dense and adipose tissues (52). The result was in accordance with previous studies that also showed the DM-based diagnosis often leads to high false negative and false positive rates due to the fact that the lesions may be obscured or hidden by the overlapping fibroglandular tissues (5, 53). The addition of DBT to DM can significantly improve the diagnostic AUC, accuracy, specificity, PPV, and NPV, and generate a similar sensitivity compared with the DM alone. This was in line with some previous reports that also indicated that breast DBT can lead to improvements in AUC and specificity by visual assessments (54, 55). This may be because the DBT can improve the lesion visibility by providing thin section tomographic images and reducing the overlap of breast tissues, and, hence, represents a clearer edge, shape, and structure of the lesion. The addition of DBT to DM did not improve the diagnostic sensitivity by visual assessments compared with DM alone as reported in an earlier study (14). The discordance may be because they performed the research with a cancer-only population. The DCE plus DWI yielded higher AUCs and sensitivities, but lower specificities than the DM plus DBT. The result was partially in line with a previous literature that also indicated that the MRI was superior to the X-ray technology in the diagnostic AUC and sensitivity, but weaker in the specificity (14, 56).

The DBT showed a similar diagnostic AUC, slightly increased specificity, and lower sensitivity compared with DCE or DWI, which was in line with a previous research that also demonstrated the inferiority of breast DBT in the sensitivity compared with MRI by visual examinations (14, 53, 57). This may be explained since the DCE can reflect the neoangiogenesis within the tumor that is associated with the growth and progression of the malignant tumor (58). While, the DWI can represent tissue microenvironments and membrane integrities through depicting the diffusivity of the tissues (59). Therefore, the MRI tends to be more sensitive than DBT or DM on tumors with higher malignant degrees. The DCE yielded higher AUC, accuracy, sensitivity, and specificity compared with DWI, which may be due to the higher resolution and the use of a contrast agent in DCE (44). We found that the addition of DBT to MRI (DBT plus DCE plus DWI) can increase the AUC and sensitivity compared with MRI alone (DCE plus DWI). This indicated that the DBT and MRI are complementary, their combination can significantly improve the predictive capabilities. While, our results were inconsistent with a previous report that showed no improvement in the diagnostic sensitivity by combing DM, DBT, DCE, and ultrasound (60). Since they involved ultrasound, direct comparisons between our study and their work was impossible.

In the clinical practice, although integrating MRI with X-rays allows the radiologists to give judgments more easily, the diagnosis still relies on subjective experiences. We selected a total of seven quantitative features as the most important predictors, three from DBT, two from DCE, and two from DWI. There were one original and six transformed features. The developed combined Rad score integrating these features significantly improved the diagnostic performance compared with any modality alone. The Original_glcm_ClusterShade feature measures the skewness and the uniformity of the gray level co-occurrence matrix within the tumor. A higher value of this feature implies a greater asymmetry about the mean and a greater heterogeneity of the lesion. We found that this feature was bigger in the malignant lesions than in the benign lesions, which suggests that a tumor with more asymmetry and complexity in the tumor texture tends to be malignant. Among the six transformed features, one belonged to the first-order and five belonged to the textural feature class. The first-order feature describes the distribution of voxel intensities in the image region. While, the textural feature quantifies the complexity of a tumor and the thickness of the texture. Our findings suggest that the tumor heterogeneity may be closely related to breast cancer, since textural features in the medical image often reflect tumor heterogeneities. The results were partially in line with previous studies that also highlighted the correlations between the textural features and breast cancer (61, 62). Our findings may explain that the proposed combined Rad score can significantly improve the diagnostic performance with regard to AUC and sensitivity than visual assessments, since most of the identified features (6 of 7) were derived from the transformed images that were generated by filtering the original images with various filters, and, thus, can hardly be understood by human.

A clinical model was built integrating age and menstruation status, and showed a lower AUC, sensitivity, and specificity than the combined Rad score. The nomogram incorporating the combined Rad score with the age and menstruation status achieved the best overall diagnostic performance compared with the combined Rad score, clinical model, and BI-RADS assessment. Decision curves demonstrated a better clinical usefulness of the nomogram with more net benefits across the majority of the range of threshold probabilities. Therefore, we suggest that our nomogram may be considered as an effective tool that can assist in decision making for the diagnosis of breast cancer. To use our nomogram, radiologists need to manually segment lesions on the DBT and MRI images for each patient, then calculate the probability of being benign or malignant. After that, clinicians can incorporate the nomogram-predicted probabilities with other clinical information to give a comprehensive decision on further examinations and treatments.

This study has limitations. First, this retrospective study had a relatively small sample size, which may cause inherent bias. Second, all data were obtained from a single hospital. Further multi-center trials are warranted to confirm the present findings. Third, our radiomic methods rely on manual segmentations of the ROIs, which were subjective and time-consuming. Future studies are needed to explore deep learning-based automatic segmentation methods on breast data.

## Conclusions

Our results showed that the DBT performed similar to DCE and DWI in terms of AUC and sensitivity, but better in specificity for detecting malignant lesions. The DBT plus DM can provide a lower AUC and sensitivity, but a higher specificity compared with DCE plus DWI. The proposed nomogram achieved the best diagnostic performance, and may help clinicians make precise decisions regarding treatments.

## Data Availability Statement

The original contributions presented in the study are included in the article/**Supplementary Material**. Further inquiries can be directed to the corresponding authors.

## Ethics Statement

All analyses of human data conducted in this study were reviewed and approved by the Institutional Review Board of the Cancer Hospital of China Medical University and in accordance with the ethical standards of the institutional and/or national research committee. Written informed consent for participation was not required for this study in accordance with the national legislation and the institutional requirements.

## Author Contributions

XJ and SN contributed to the study concepts and manuscript preparation. SN and XW contributed to the study design. NZ and GL contributed to the data acquisition. SN and NZ contributed to the quality control of the data and algorithms. XJ and XW contributed to the data analysis and interpretation. YL and E-NC contributed to the statistical analysis. XJ, YD and YK contributed to the manuscript review. All authors contributed to the article and approved the submitted version.

## Funding

The study was funded by the Key Program of Ministry of Science and Technology of China (2017YFC1309100), Climbing Fund of National Cancer Center (NCC201806B011), Shenyang Municipal Science and Technology Project (F16-206-9-23), National Natural Science Foundation of China (81872363), Support program of youth science and technology innovation talents of Shenyang City (RC180269), Major Technology Plan Project of Shenyang (17-230-9-07), Supporting Fund for Big data in Health Care (HMB201903101), Special foundation for the central government guides the development of local science and technology of Liaoning Province (2018416029), China National Natural Science Foundation (31770147), and Medical-Engineering Joint Fund for Cancer Hospital of China Medical University and Dalian University of technology (LD202029).

## Conflict of Interest

The authors declare that the research was conducted in the absence of any commercial or financial relationships that could be construed as a potential conflict of interest.

## Publisher’s Note

All claims expressed in this article are solely those of the authors and do not necessarily represent those of their affiliated organizations, or those of the publisher, the editors and the reviewers. Any product that may be evaluated in this article, or claim that may be made by its manufacturer, is not guaranteed or endorsed by the publisher.
