# Supplementary material for: Radiomic Evaluations of the Diagnostic Performance of DM, DBT, DCE MRI, DWI, and Their Combination for the Diagnosisof Breast Cancer
Source: Front Oncol. 2021 Sep 10;11:725922. doi: 10.3389/fonc.2021.725922 (PMC8461299; doi:10.3389/fonc.2021.725922)
Supplement: Supplementary file 1 [file DataSheet_1.docx]

Supplementary Material

Table S1. Digital breast tomosynthesis parameters

| Parameter | Value |
| --- | --- |
| Intersection spacing (mm) | 1 |
| voltage range (kV) | 20.0 - 49.0 |
| nominal power (kW) | 3.0 |
| current time range (mAs) | 300 - 400 |
| scanning time (s) | 4.0 |
| reconstruction time (s) | 2.0 - 5.0 |
| pixel size (μm) | 70 |

Table S2. Brest MRI parameters

| Parameter | DCE | DWI |
| --- | --- | --- |
| TR (ms) /TE (ms) /TI (ms) | 6.2 /3.0 /13 | 5000 /64 /0 |
| Slice thickness (mm) | 3.2 | 6 |
| Slice gap (mm) | 3.2 | 7.5 |
| Field of view (mm) | 360 | 240 |
| Matrix size | 350 × 350 | 128 × 128 |
| b-value (s/mm^2^) | - | 800 |
| Flip angle (deg) | 10 | 90 |

*DCE*, dynamic contrast enhanced; *DWI*, diffusion-weighted imaging; *TR*, repetition time; *TE*, echo time; *TI*, inversion time

S3. The details about rad score.

The rad score was generated using the least absolute shrinkage and selection operator (LASSO) algorithm and stepwise multivariate logistic regression with the Akaike’s Information Criterion (AIC) as the stopping rule. The value of rad score can be positive or negative with each patient which represents the malignancies or benign tumors predicted by the combined radiomic model.
